# CRISPR Screens Identify *Toxoplasma* Genes That Determine Parasite Fitness in Interferon Gamma-Stimulated Human Cells

**DOI:** 10.1128/mbio.00060-23

**Published:** 2023-03-14

**Authors:** Shruthi Krishnamurthy, Parag Maru, Yifan Wang, Mebratu A. Bitew, Debanjan Mukhopadhyay, Yoshiki Yamaryo-Botté, Tatiana C. Paredes-Santos, Lamba O. Sangaré, Christopher Swale, Cyrille Y. Botté, Jeroen P. J. Saeij

**Affiliations:** a Department of Pathology, Microbiology and Immunology, School of Veterinary Medicine, University of California Davis, Davis, California, USA; b Apicolipid Team, Institute for Advanced Biosciences, CNRS UMR5309, INSERM U1209, Université Grenoble Alpes, Batiment Jean Roget, Grenoble, France; c Team Host-Pathogen Interactions and Immunity to Infection, Institute for Advanced Biosciences (IAB), INSERM U1209, CNRS UMR5309, University Grenoble Alpes, Grenoble, France; Albert Einstein College of Medicine

**Keywords:** CRISPR screen, Toxoplasma gondii, effector functions, host-pathogen interactions, interferons

## Abstract

*Toxoplasma* virulence depends on its ability to evade or survive the toxoplasmacidal mechanisms induced by interferon gamma (IFNγ). While many *Toxoplasma* genes involved in the evasion of the murine IFNγ response have been identified, genes required to survive the human IFNγ response are largely unknown. In this study, we used a genome-wide loss-of-function screen to identify *Toxoplasma* genes important for parasite fitness in IFNγ-stimulated primary human fibroblasts. We generated gene knockouts for the top six hits from the screen and confirmed their importance for parasite growth in IFNγ-stimulated human fibroblasts. Of these six genes, three have homology to GRA32, localize to dense granules, and coimmunoprecipitate with each other and GRA32, suggesting they might form a complex. Deletion of individual members of this complex leads to early parasite egress in IFNγ-stimulated cells. Thus, prevention of early egress is an important *Toxoplasma* fitness determinant in IFNγ-stimulated human cells.

## INTRODUCTION

*Toxoplasma* can infect a broad range of warm-blooded animals, including humans (1) and is the second leading cause of foodborne illness in the USA (2). In immunosuppressed individuals, reactivation of tissue cysts within the heart, brain and skeletal muscle can lead to serious myocardial (3–6) and neurological complications (3, 7). Infection during pregnancy can lead to birth defects or cause abortion of the developing fetus (8). Current drugs cause severe side effects (9) and there is a lack of preventative vaccines, therefore, new drug targets are constantly needed. Identifying *Toxoplasma* genes that determine survival during the immune response in humans would lay the much-needed groundwork for identifying new drug targets.

*Toxoplasma* clonal types I, II, III, and XII are predominant in North America (10, 11) and these strains vary in virulence, with the type I strain being one of the most virulent strains in inbred house mice with a lethal dose (LD_100_) <10 (12). The mode of recognition and clearance of *Toxoplasma* is distinct in humans and mice. In mice, Toll-like receptors 11 and 12 (TLR11 and TLR12) in dendritic cells detect *Toxoplasma* profilin, leading to the secretion of interleukin 12, which can subsequently induce interferon gamma (IFNγ) secretion by natural killer cells and T cells (13, 14). IFNγ induces a variety of toxoplasmacidal mechanisms in both hematopoietic and nonhematopoietic cells. In mice, these toxoplasmacidal mechanisms are dominated by induction of the immunity related GTPases (IRGs) and Guanylate binding proteins (GBPs) that destroy the parasitophorous vacuole (PV) the parasite lives in and subsequently the parasite itself (15–18). Paradoxically, humans lack functional TLR11/12 and IFNγ-inducible IRGs but are relatively resistant to *Toxoplasma* (19). The IFNγ-induced toxoplasmacidal mechanism depends on the human cell type and the infecting *Toxoplasma* strain (20). For example, infection of IFNγ-stimulated human umbilical vein endothelial cells (HUVEC) with the type II, but not the type I, strain leads to ubiquitination and subsequent destruction of the PV by lysosomal fusion (21), whereas infection of IFNγ-stimulated HeLa cells with type II or type III, but not type I, strains causes growth stunting by noncanonical autophagy (22). IFNγ induces atypical apoptotic cell death in human macrophages infected with type I and type II strains. Cell death is mediated by Apoptosis-associated Speck-like protein (ASC) and caspase 8 upon GBP-1 and absent in melanoma 2 (AIM2) mediated detection of *Toxoplasma* (23). IFNγ can also affect the availability of nutrients for *Toxoplasma* (24, 25) including those that *Toxoplasma* is an auxotroph for such as l-tryptophan and cholesterol (26). IFNγ upregulates tryptophan catabolism *via* induction of the enzyme Indoleamine-2,3-dioxygenase (IDO), which inhibits growth of *Toxoplasma* in certain cell types (27–34). We previously published that when primary human foreskin fibroblasts (HFFs) were stimulated with IFNγ, and subsequently infected with the type I RH strain, it resulted in cell death along with early parasite egress without replication (31). By overexpressing IFNγ-stimulated host genes, it was recently determined that Retinoic acid receptor responder protein 3 (RARRES3) induces early parasite egress in multiple human cell types (35).

*Toxoplasma* modulates the host immune response by secreting proteins that reside in the dense granules (GRAs) and rhoptries (ROPs). While rhoptry contents are released during invasion, GRAs are secreted once the parasite establishes successful infection within the host cell with the formation of the PV. ROPs and GRAs together ensure parasite survival within the PV by modifying the PV membrane (PVM), by altering host signaling pathways, and by preventing PVM destruction by IRGs/GBPs (36, 37). In mice, *Toxoplasma* ROP18, ROP5, ROP17, ROP16, ROP54, GRA7, and GRA60 are important to block IRG/GBP-mediated destruction of the PV (38–45). We recently reported that type II strain growth is restricted in IFNγ-activated HFFs via GRA15-mediated recruitment of ubiquitin ligases, including TNF associated receptor factor (TRAF)2 and TRAF6, to the PVM, which enhances recruitment of ubiquitin receptors (p62/NDP52) and ubiquitin-like molecules (LC3B, GABARAP) and eventual PV destruction by lysosomes (46). It was recently shown that the ubiquitin ligase RNF213 is also recruited to the PVM in human cells where it mediates PV ubiquitination, recruitment of ubiquitin receptors, and parasite restriction (47). An example of a GRA that is conserved across clonal types is TgIST (*Toxoplasma* Inhibitor of STAT1 Transcriptional activity), which localizes to the host cell nucleus and recruits a chromatin repressor that inhibits STAT1 transcriptional activity and thereby IFNγ-inducible toxoplasmacidal mechanisms (48, 49). TgNSM (NCoR/SMRT modulator) further enhances the ability of the NCoR/SMRT complex to inhibit inflammatory gene expression. TgIST together with TgNSM can block HFF necroptosis upon IFNγ stimulation by blocking the expression of protein kinase R (PKR) and mixed lineage kinase domain-like pseudokinase (MLKL), which are critical for necroptosis (50, 51). Although TgIST and TgNSM can block IFNγ signaling, what *Toxoplasma* genes are needed for survival in human cells that have been prestimulated with IFNγ is unknown.

Here, we performed a genome-wide loss-of-function screen in the *Toxoplasma* type I RH strain and identified multiple parasite genes that determine fitness in IFNγ-stimulated HFFs. We further characterized six of these genes, five of which encode GRAs (TGGT1_217680/GRA57 [52], TGGT1_272460/GRA72, TGGT1_249990/GRA70, TGGT1_309600/GRA71, and TGGT1_320490/GRA66 [53]) and confirmed that they play a role in resistance to IFNγ-mediated parasite growth inhibition in HFFs. GRA57, GRA70, and GRA71 coimmunoprecipitated with each other and GRA32 suggesting they might form a complex. Infection of IFNγ-stimulated HFFs with parasites containing a deletion in individual members of this putative complex led to enhanced host cell death, which was prevented when parasite egress was inhibited. Thus, prevention of early parasite egress in IFNγ-stimulated human cells is a major parasite fitness determinant.

## RESULTS

### Genome-wide loss-of-function screen identifies *Toxoplasma* genes that determine fitness in IFNγ-stimulated human fibroblasts.

**FIG 1:**
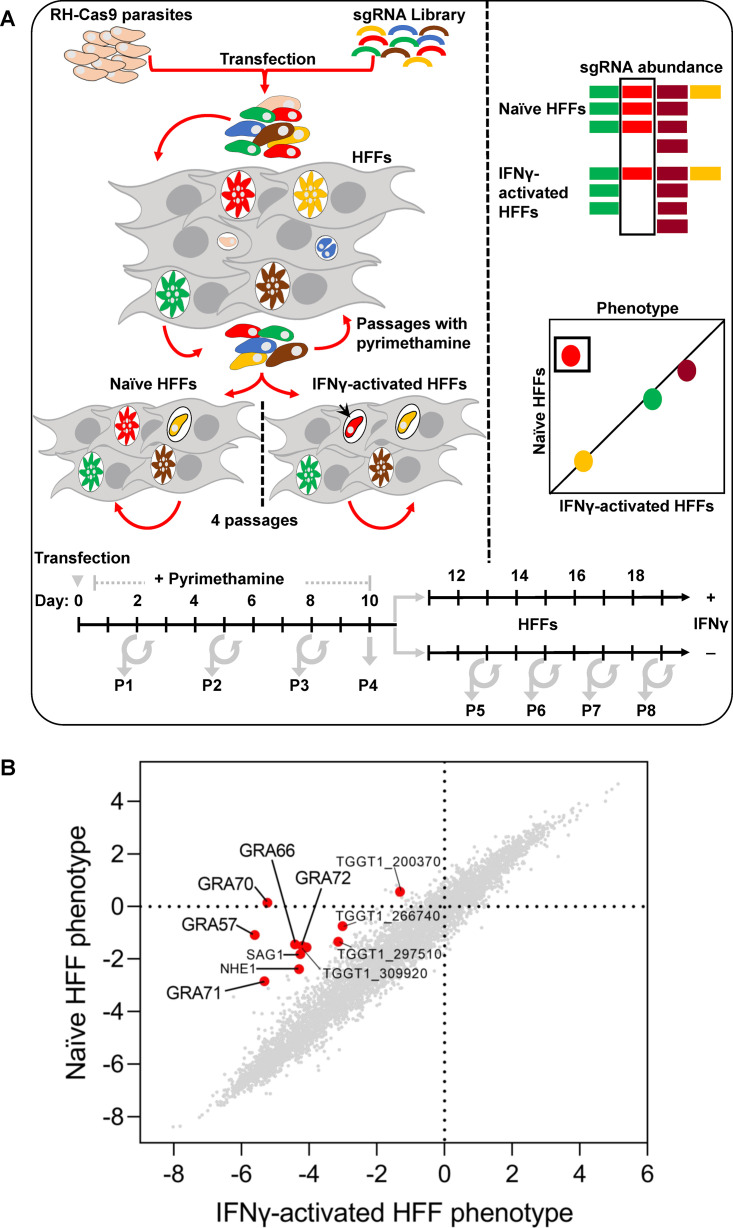
*Toxoplasma* genome-wide loss-of-function screen in naive or IFNγ-activated HFFs. (A) Screening procedure. RH-Cas9 parasites were transfected with linearized plasmids containing 10 sgRNAs against every *Toxoplasma* gene. Transfected parasites were passaged for 4 to 5 passages in HFFs under pyrimethamine selection to remove nontransfected parasites and parasites that integrated plasmids with sgRNAs targeting parasite genes important for fitness in HFFs. Subsequently, the pool of mutant parasites was passaged 4 times in naive or IFNγ-stimulated HFFs. (B) The sgRNA abundance at different passages, determined by Illumina sequencing, was used for calculating phenotype scores and identifying genes that confer fitness specifically in IFNγ-activated HFFs. Genes shown in Table 1 are indicated with a red dot.

To identify *Toxoplasma* genes that determine fitness in IFNγ-stimulated human cells, we performed a genome-wide loss-of-function screen. We used the RH type I parasite strain expressing Cas9 (RH-Cas9), which has already been implemented with success in other *Toxoplasma* loss-of-function screens (25, 54–56). We generated a *Toxoplasma* mutant pool by transfecting RH-Cas9 parasites (57) with a library of sgRNAs containing 10 guides for each of the 8,156 *Toxoplasma* genes. This mutant pool was grown for 4 to 5 passages in HFFs to enrich for mutants without a general fitness defect in naive HFFs (54, 55). We subsequently passaged the pool of parasite mutants an additional four times in naive or IFNγ-stimulated HFFs (Fig. 1A) or IFNγ-stimulated murine bone marrow-derived macrophages (BMDMs) (58). We amplified, sequenced, and quantified the sgRNAs from the input library, passage 4 to 5 in naive HFFs, and after the additional 4 passages (total passage 8/9) in naive or IFNγ-stimulated HFFs. We calculated the average log_2_ fold change in sgRNA abundance targeting a specific gene in these samples relative to the input library and defined it as the phenotype score for that gene. As previously described (58), there was a high correlation (*r* = 0.81 ± 0.03, mean ± SEM, *n* = 3) between our mean naive HFF passage 4 phenotype scores and previously published phenotype scores (54). By subtracting the naive HFF phenotype score from the IFNγ-stimulated HFF phenotype score at passage 8/9, we identified 54 parasite genes that specifically determined fitness in IFNγ-stimulated HFF (*P* < 0.05; IFNγ-stimulated – naive HFF phenotype score <–1; IFNγ-stimulated HFF phenotype score <–1) with a large effect size (Cohen’s *d* ≥ 0.8) (Table S3). Gene set enrichment analysis (GSEA) of these genes indicated enrichment in protein farnesyltransferase activity, steroid biosynthesis, and glutathione metabolism, among others (Table S4).

10.1128/mbio.00060-23.3 TABLE S3 The Phenotype_Scores sheet contains the phenotype scores and *P* values (determined by MAGeCK) for each parasite gene in naïve and IFNγ-stimulated HFFs for each of the three experiments performed. It also contains the phenotype scores in IFNγ-stimulated BMDMs and *P* values (determined by MAGeCK) comparing IFNγ-stimulated HFFs to IFNγ-stimulated BMDMs. Download Table S3, XLSX file, 4.1 MB. Copyright © 2023 Krishnamurthy et al. 2023 Krishnamurthy et al. https://creativecommons.org/licenses/by/4.0/ This content is distributed under the terms of the Creative Commons Attribution 4.0 International license.

10.1128/mbio.00060-23.4 TABLE S4 The 54 hits listed in Table S3 were analyzed for enrichment in functional annotation (GO and metabolic pathways) using tools available in ToxoDB. Download Table S4, XLSX file, 0.01 MB. Copyright © 2023 Krishnamurthy et al. 2023 Krishnamurthy et al. https://creativecommons.org/licenses/by/4.0/ This content is distributed under the terms of the Creative Commons Attribution 4.0 International license.

Eleven high-confidence candidate genes are presented in Table 1, five of which are predicted dense granule proteins by localization of organelle proteins by isotope tagging (LOPIT) (59) but only GRA57 (TGGT1_217680) and GRA66 (TGGT1_320490) have been confirmed to be GRAs that localize to the PV (52, 53). TGGT1_249990, GRA57 and TGGT1_309600 have sequence similarity to GRA32 (TGGT1_212300) and each other. TGGT1_297510 and TGGT1_200370 are predicted to encode the alpha and beta subunit of a farnesyltransferase and these two genes were also predicted to determine fitness in IFNγ-stimulated murine macrophages (58). TGGT1_266740 is an RNA recognition motif-containing protein with high homology to the polyadenylate-binding protein RBP47B (BLASTP E value, 2 × 10^−37^) a key component of stress granules (60), which have been shown to be important for extracellular *Toxoplasma* to survive and remain infective (61). TGGT1_259200B is the Na+/H+ exchanger NHE1, which regulates ionophore-induced egress (62). IFNγ has been shown to induce premature parasite egress (31, 35). The *Toxoplasma* surface antigen SAG1 (TGGT1_233460) determined fitness in both this screen and in IFNγ-stimulated murine BMDM (58). *GRA66* encodes a predicted *N*-acylphosphatidylethanolamine (NAPE)-specific phospholipase D (NAPE-PLD) (HHpred [63] with human NAPE-PLD, E value 4.3e^−46^), which are part of the metallo-β-lactamase superfamily and GRA66 contains the conserved zinc-binding motif HxHxDH (64). NAPE-PLDs can theoretically convert NAPE into fatty acid ethanolamides (FAE) and phosphatidic acid (65–67). Phosphatidic acid is known to be a pivotal lipid class that, when generated in the PV lumen by the action of diacylglycerol kinase 2 (TgDGK2), is involved in regulating microneme secretion for *Toxoplasma* invasion and egress (68–70). Six of the genes in Table 1 were also significant when comparing their phenotype scores in IFNγ-stimulated HFFs versus IFNγ-stimulated BMDM (58). This suggests these parasite genes determine fitness specifically in IFNγ-stimulated human cells although a cell type specific role (fibroblasts versus macrophages) cannot be excluded.

**TABLE 1:** Top hits from loss-of-function screen in IFNγ-stimulated HFFs^*a*^

ToxoDB_ID	Description	Localization	IFNγ vs naive HFFs phenotype^*b*^	IFNγ vs naive HFFs *P* value	Cohen’s *d*	IFNγ vs naive murine BMDM hit	IFNγ HFFs vs IFNγ BMDM phenotype^*c*^	IFNγ HFFs vs IFNγ BMDM *P* value
TGGT1_249990	Hypothetical protein/GRA70	Dense granules	−5.4 ± 1.2	7.7E-7	9.8	No	−4.2	0.004
TGGT1_217680	GRA57	Dense granules	−4.5 ± 1.1	7.7E-7	9.1	No	−2.1	6.2E-04
TGGT1_320490	*N*-acylphosphatidylethanolamine-hydrolyzing phospholipase D family protein/GRA66	Dense granules	−3.0 ± 0.5	5.4E-6	6.9	No	−1.2	0.03
TGGT1_272460	Hypothetical protein/GRA72	Dense granules	−2.7 ± 1.0	0.002	4.9	No	0.5	0.06
TGGT1_309600	Hypothetical protein/GRA71	Dense granules	−2.5 ± 1.1	0.001	4.8	No	−2.3	0.005
TGGT1_309920	Hypothetical protein	Mitochondrion − membranes	−2.5 ± 0.8	0.03	2.5	No	−3.0	0.03
TGGT1_233460	SRS29B (SAG1)	PM − peripheral 1	−2.5 ± 1.8	4.2E-4	3.0	Yes	−0.0	0.23
TGGT1_266740	Predicted polyadenylate-binding protein RBP47B		−2.3 ± 1.5	0.005	3.4	No	−1.3	0.01
TGGT1_259200B	Na+/H+ exchanger NHE1	PM	−1.9 ± 1.5	0.003	2.5	No	−0.3	0.25
TGGT1_200370	Predicted farnesyl transferase beta subunit		−1.9 ± 0.5	0.002	3.9	Yes	1.3	0.2
TGGT1_297510	Predicted farnesyl transferase alpha subunit		−1.8 ± 0.7	0.02	4.8	Yes	1.7	0.38

a From 54 parasite genes that specifically determined fitness in IFNγ-stimulated HFF (difference in the average phenotype scores between IFNγ-stimulated and naive HFF <−1 [*P* < 0.05], average IFNγ-stimulated HFF phenotype < −1) with a large effect size (Cohen’s *d* ≥ 0.8), we selected 11 hits that had negative IFNγ-stimulated HFF phenotype scores and lower phenotype scores in IFNγ-stimulated versus naive HFFs in all three screens and *P* < 0.05 (MAGeCK) in at least 2 out of the 3 screens with a Cohen’s *d* ≥ 1. Localization prediction is based on LOPIT data in ToxoDB. *P* values were calculated with MAGeCK using the raw reads from the three screens.

b IFNγ versus naive HFFs phenotype column shows the average difference between phenotype scores in IFNγ-stimulated HFFs and naive HFFs (±SD).

c IFNγ HFFs versus IFNγ BMDM phenotype column shows the average difference between phenotype scores in IFNγ-stimulated HFFs and IFNγ-stimulated BMDMs.

Other notable hits (Table S3) include the following: TGGT1_204100, which encodes TgIF2K-C, a GCN2-related kinase that was previously shown to be important for the parasite’s response to nutrient (glutamine) starvation (71); GRA23 (TGGT1_297880), which together with GRA17 forms pores in the PVM that could mediate the uptake of small nutrients from the host cytoplasm (72); TGGT1_269035, which encodes a nucleoside diphosphate kinase involved in purine metabolism; Acyl-CoA:cholesterol acyltransferase alpha (ACAT1-alpha, TGGT1_263710), which plays an important role in the storage of host-derived cholesterol in lipid bodies (73); Rhoptry Apical Surface Protein 1 (TgRASP1, TGGT1_235130), which contains a C2 and Pleckstrin Homology (PH) domain that have been shown to be involved in binding to lipids and was recently shown to be essential for rhoptry discharge and invasion (74); Peroxiredoxin 3 (PRX3, TGGT1_230410), which was recently identified in a genome-wide CRISPR screen to be important for resistance against oxidative stress induced by hydrogen peroxide (75); TGGT1_213620, which has homology to ADCK3/ABC1/UbiB (HHpred, E value, 5.9 × 10^−51^), which belong to the UbiB protein kinase-like family and are involved in the biosynthesis of isoprenoid lipids like coenzyme Q (also known as ubiquinone) that can function as antioxidants against radicals produced in membranes; TgAlba2 (TGGT1_218820) an RNA-binding protein, which has been shown to localize to stress granules (76); *Toxoplasma* ER-resident calcium binding protein (TgERC, TGGT1_229480), an orthologue of Plasmodium falciparum (Pf)ERC, which was shown to be important in regulating parasite egress (77); ROP9 (TGGT1_243730), which has been shown to affect invasion and egress (78). Thus, multiple parasite genes that determine fitness in IFNγ-stimulated HFFs seem to be involved in regulating parasite egress, the response to oxidative stress, and parasite (lipid) metabolism. Of the parasite genes that affect GRA export, only ROP17 (79) was in our list of hits. Although MYR3 (80) and GRA45 (58, 81) did not meet our stringent criteria to be included in the list of hits, they were significant hits in three and two of the screens, respectively, and were in the top 2 percentile of hits (Table S3). Neither MYR1, MYR2, and MYR4, nor any of the GRAs secreted beyond the vacuole were significant hits in the three screens we performed, indicating that GRA export does not contribute to parasite fitness in HFFs that have been prestimulated with IFNγ.

### Validation of *Toxoplasma* candidate genes that determine fitness in IFNγ-stimulated HFFs.

**FIG 2:**
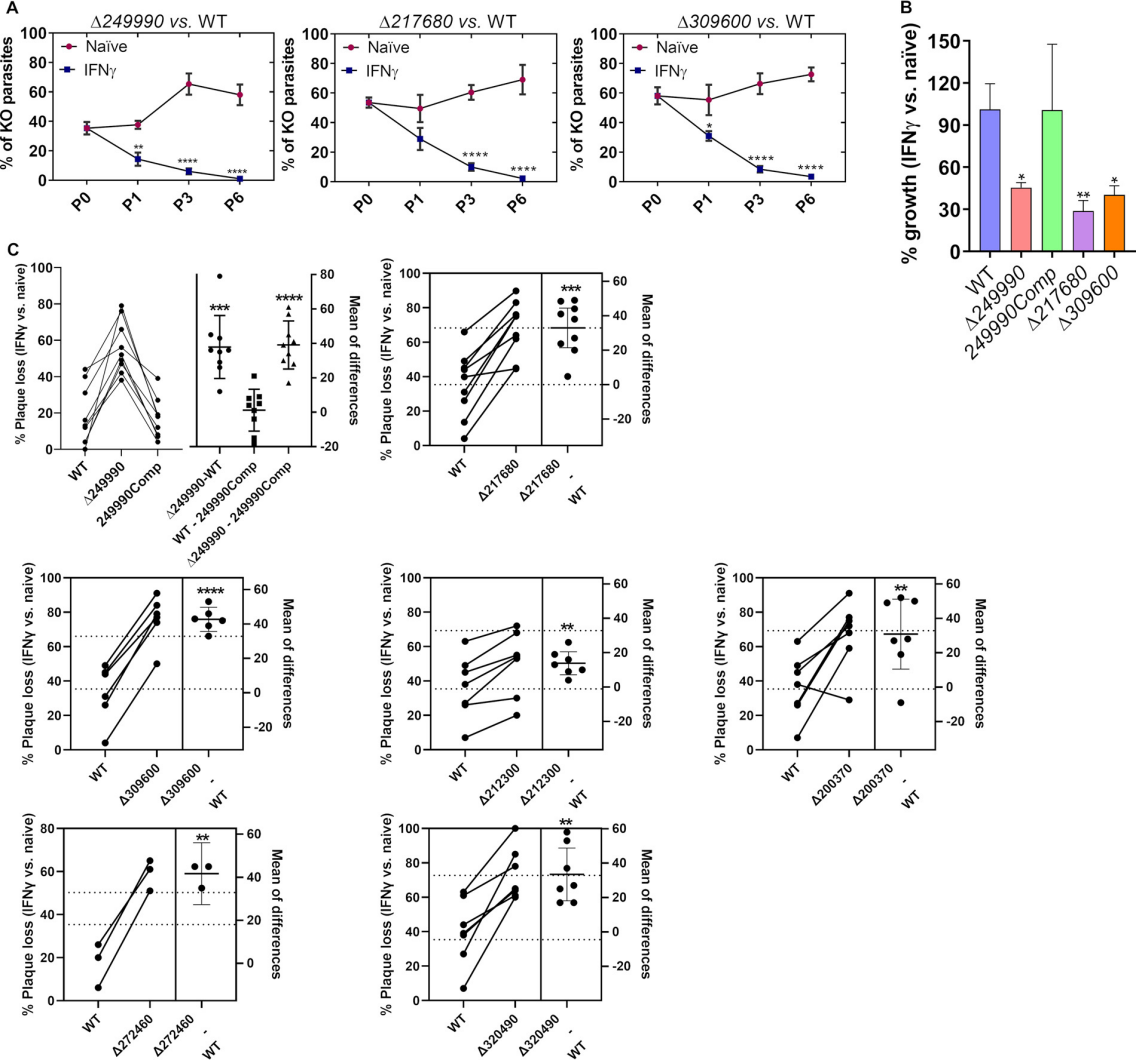
Six of the top candidate genes identified in the CRISPR screen determine fitness in IFNγ-stimulated HFFs. (A) Indicated GFP-expressing knockout (KO) strains (numbers shown are TGGT1_ T. gondii gene ID numbers) generated in an RH (type I) luciferase-expressing background were mixed in a 1:1 ratio with luciferase-expressing wild-type (WT) parasites and passaged for 6 passages on naive or IFNγ-stimulated HFFs. The percentage of KO parasites was determined at passage 0, 1, 3, and 6 by plaque assays. Two-way ANOVA followed by Sidak’s multiple-comparison test was used for statistical analysis. (B) Naive or IFNγ-stimulated (20 U/mL) HFFs were infected with indicated parasite strains (249990Comp is Δ249990 complemented with a WT copy of 249990) and 24 hpi, parasite luciferase was quantified with a luminometer. Indicated is the percentage of luciferase in IFNγ-stimulated HFFs compared to unstimulated HFFs for each strain. Two-way ANOVA followed by Dunnett’s multiple-comparison test was used for statistical analysis. (C) Percentage decrease in number of lysis plaques formed in IFNγ-stimulated versus naive HFFs is plotted for indicated strains. On the right of each graph, the mean difference ±SD between KO and WT is indicated. Each data point is from a separate biological experiment (but some WT data points are shared among graphs from when multiple KO parasites were compared at the same time to WT). Paired Student's *t* test was used for the comparisons between WT and KO parasites, while ANOVA was used to compare WT, Δ*249990*, and 249990Comp. *, *P* < 0.05; **, *P* < 0.01; ***, *P* < 0.001; ****, *P* < 0.0001 versus WT.

We generated individual knockouts for several of the candidate genes (Fig. S1) in the RH luciferase background. We performed a growth competition assay between the wild type and three of the knockout parasites during 6 serial passages in naive and IFNγ-stimulated HFFs. Δ*249990*, Δ*gra57*, and Δ*309600* were outcompeted by wild-type parasites specifically in IFNγ-stimulated HFFs (Fig. 2A). We complemented the Δ*249990* parasite strain by randomly integrating 249990-HA expressed from its endogenous promoter (Fig. S2). We infected naive or IFNγ-stimulated HFFs with wild-type, Δ*249990*, Δ*gra57*, Δ*309600*, and the Δ*249990 + *249990-HA parasites and determined relative growth by measuring luciferase. The knockout parasites had a significant reduction in growth relative to wild-type parasites in IFNγ-stimulated but not naive HFFs (Fig. 2B). The Δ*249990 + *249990-HA strain was significantly more resistant to IFNγ-mediated growth inhibition compared to the Δ*249990* strain, confirming that the IFNγ-mediated growth inhibition observed in Δ*249990* parasites was due to deletion of *TGGT1_249990*. To confirm additional hits from our screen we also generated knockouts for *TGGT1_200370* and *GRA66*, and since three of our top candidate hits have homology to GRA32, we also generated Δ*gra32* parasites (Fig. S1). Plaque assays performed on naive or IFNγ-stimulated HFFs showed that compared to wild-type parasites, Δ*249990*, Δ*gra57*, Δ*309600*, Δ*gra32*, Δ*200370*, and Δ*gra66* parasites had a significantly increased percent reduction of plaques formed in IFNγ-stimulated versus naive HFFs (Fig. 2C). Overall, these results indicate that the genes identified by our screen determine fitness specifically in IFNγ-stimulated HFFs.

10.1128/mbio.00060-23.6 FIG S1 Screening individual knockout parasites generated by sgRNA-directed CRISPR/Cas9 genome editing. (A) Schematic of HXGPRT or DHFR repair template integrating either in the forward or reverse orientation after sgRNA directed Cas9 double-stranded DNA cleavage at the genomic locus of the gene of interest (GOI). Primers used to screen clones of knockout parasites in either forward or reverse orientation are indicated along with the image of the amplified products below. (B to H) Knockout parasites were generated keeping the GFP intact in the repair template generated from the restriction digest linearized pTKO plasmid. The schematic indicates the sites at which the primers for screening knockout parasites anneal and images of the gels showing the amplicons for the indicated knockout parasites are shown. For all knockout parasites, the genomic locus was disrupted as seen in the PCR with p1+p3 or p3+p2. 1 kb plus ladder from Invitrogen was used for all 1% agarose gels. Download FIG S1, TIF file, 5.4 MB. Copyright © 2023 Krishnamurthy et al. 2023 Krishnamurthy et al. https://creativecommons.org/licenses/by/4.0/ This content is distributed under the terms of the Creative Commons Attribution 4.0 International license.

**FIG 3:**
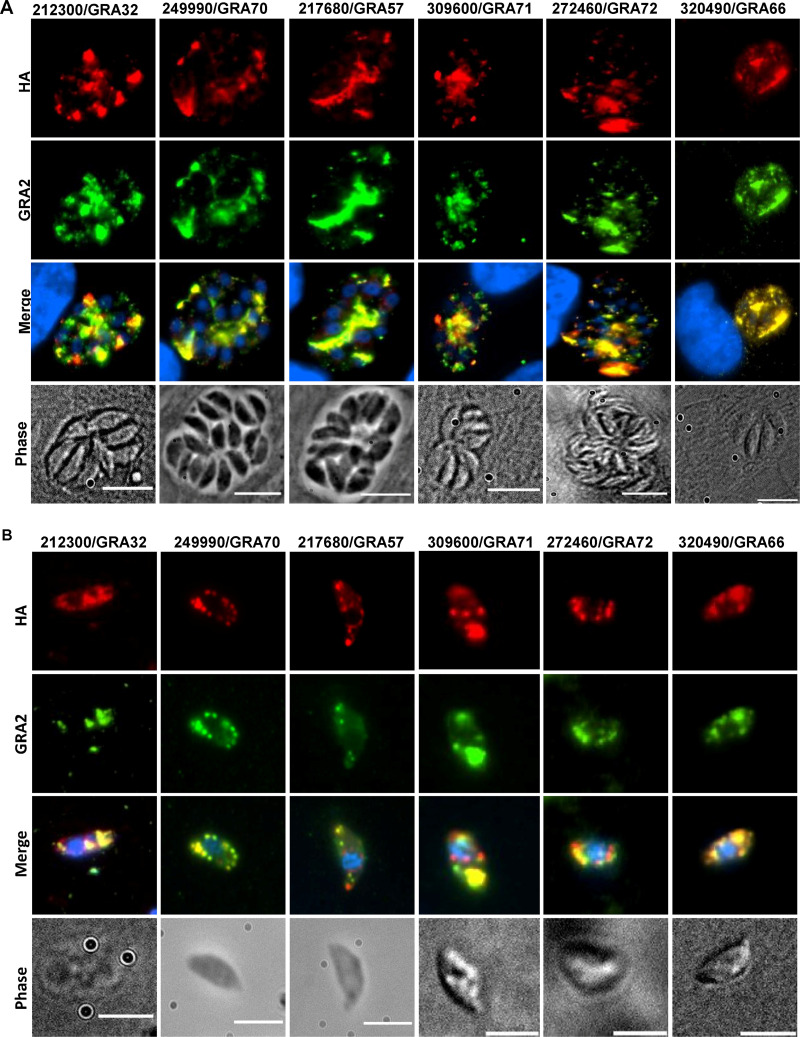
Five hits from the screen are dense granule proteins. (A) IFA using intracellular parasites indicating localization to the parasitophorous vacuole (PV) lumen and, to a certain extent, to the PV membrane and colocalization with GRA2. Images are scaled at 10 μm. (B) IFA using extracellular parasites showing localization to dense granules, as indicated by colocalization with GRA2. Images are scaled at 5 μm.

10.1128/mbio.00060-23.7 FIG S2 Generation of complemented parasites. RHΔ*249990* parasites were complemented with a vector containing the TGGT1_249990 gene with a C-terminal 3xHA tag and its putative promoter as described in Materials and Methods. The immunofluorescence image shows that the HA signal (red) is mostly in the PV lumen similar to what is observed in endogenously tagged TGGT1_249990 (Fig. 3). Download FIG S2, TIF file, 2.6 MB. Copyright © 2023 Krishnamurthy et al. 2023 Krishnamurthy et al. https://creativecommons.org/licenses/by/4.0/ This content is distributed under the terms of the Creative Commons Attribution 4.0 International license.

### Localization of candidate genes products.

We endogenously tagged *TGGT1_249990*, *GRA57*, *TGGT1_309600*, *GRA32*, *TGGT1_272460*, and *GRA66* at the C terminus with the 3xHA epitope in the RHΔ*ku80*Δ*hxgprt* strain. In intracellular parasites, they localized to the PV lumen and partially to the PV membrane and colocalized with GRA2 (Fig. 3A). In extracellular parasites (Fig. 3B) they colocalized with GRA2 within dense granules. The Δ*249990 *+ 249990-HA parasite strain had a similar localization of TGGT1_249990 as the endogenously epitope-tagged strain (Fig. S2). We therefore named TGGT1_249990 GRA70, TGGT1_309600 GRA71, and TGGT1_272460 GRA72.

### GRA32, GRA57, GRA70, and GRA71 coimmunoprecipitate with each other.

To identify proteins that could interact with GRA70 and GRA57, we immunoprecipitated GRA70 and GRA57 from lysate isolated from HFFs infected with the C-terminally 3xHA-tagged strains using beads containing HA antibodies. As a control, we used immunoprecipitation data from GRA35, GRA15, and the GRA TGGT1_263560. GRA70 and GRA57 both pulled down GRA71, GRA32, and each other, suggesting GRA70, GRA57, GRA71, and GRA32 interact directly or indirectly with each other and may function in a complex (Table 2). GRA66, encoded by one of the other candidate genes from the screen (Table 1), was immunoprecipitated with GRA57. Other notable proteins that immunoprecipitated with GRA57 were: calcium-dependent protein kinase (CDPK)1, which has been shown to be important for microneme secretion and egress/invasion (82); TGGT1_252430, a protein that contains a StAR-related lipid-transfer (START) domain that is most similar to the START domain of STARD4/5/6, which are proteins involved in cholesterol transport (83); subunits from the mitochondrial ATP synthase; TgMyoF, which is involved in dense granule and other organelle trafficking (84); and ROP5.

**TABLE 2:** Immunoprecipitation of GRA70 and GRA57^*a*^

ToxoDB_ID^*b*^	Localization	Description	GRA70	GRA57	263560	GRA35	GRA15
**TGME49_217680**	Dense granules	GRA57	43	169	1	1	9
**TGME49_309600**	Dense granules	Hypothetical protein /GRA71	15	70	0	0	8
**TGME49_249990**	Dense granules	Putative microtubule-binding protein/GRA70	71	56	0	0	7
**TGME49_212300**	Dense granules	GRA32	79	22	0	0	4
TGME49_279100	Dense granules	MAF1/GRA67	0	11	0	0	0
**TGME49_320490**	Dense granules	*N*-acylphosphatidylethanolamine-hydrolyzing phospholipase D/ GRA66	0	9	0	0	1
TGME49_311720	Er 2	Chaperonin protein BiP	9	72	3	2	14
TGME49_249900	Mitochondrion − membranes	Putative adenine nucleotide translocator	0	12	0	2	2
TGME49_204400	Mitochondrion − membranes	TgATPα	0	9	0	0	0
TGME49_261950	Mitochondrion − membranes	TgATPβ	0	7	0	0	0
TGME49_278870	PM − peripheral 2	TgMyoF	3	9	2	1	0
TGME49_252430	PM − peripheral 2	Putative START-2 domain protein	0	7	0	1	1
TGME49_301440	Peripheral/cytosol/nucleus	CDPK1	0	13	0	0	0
TGVEG_442220	Rhoptries 1	ROP5	0	11	0	0	0

a Listed are the number of unique peptides that were detected by mass-spectrometry after immunoprecipitation of 3xHA-tagged GRA70 or GRA57 with anti-HA magnetic beads.

b *Toxoplasma* proteins predicted to be secretory proteins or associated with membranes and that had at least 7 unique peptides and at least a 4-fold enrichment compared to immunoprecipitations of the control proteins (GRA35, GRA15, and the GRA TGGT1_263560). Proteins listed in bold were identified as genes that determine fitness in IFNγ-stimulated HFFs (Table 1). The entire data set is presented in Table S5.

10.1128/mbio.00060-23.5 TABLE S5 Contains the total unique peptide and spectrum counts for the immunoprecipitations of GRA70, GRA57, GRA35, GRA15 and TGGT1_263560. Download Table S5, XLSX file, 0.1 MB. Copyright © 2023 Krishnamurthy et al. 2023 Krishnamurthy et al. https://creativecommons.org/licenses/by/4.0/ This content is distributed under the terms of the Creative Commons Attribution 4.0 International license.

### GRA32, GRA57, GRA70, and GRA71 have similar structures and are conserved in many coccidia.

Since our immunoprecipitation results indicated that GRA32, GRA57, GRA70, and GRA71 might function in the same pathway through direct or indirect protein interactions, we chose to focus on determining the mechanism by which they affect parasite fitness in IFNγ-stimulated HFFs. The genes encoding these proteins are highly expressed in all *Toxoplasma* life stages (85, 86) and have orthologues in all coccidian species belonging to the *Sarcocystidae* (Neospora caninum, Hammondia hammondi, *Cystoisospora suis*, *Sarcocystis* spp.) and *Eimeriidae* (*Eimeria* spp. and *Cyclospora* spp.) (EupathDB.org [87]). Most GRAs are only conserved within the *Toxoplasmatinae*, suggesting that these GRAs have a conserved function in these different parasite species. The ratio of nonsynonymous and synonymous substitutions (dN/dS) of *GRA70* (dN/dS = 0.52) and *GRA71* (dN/dS = 0.48) indicate these genes are under purifying selection, while *GRA32* (dN/dS = 1) seems to be under neutral selection and *GRA57* (dN/dS = 1.63) under positive selection. Although ToxoDB does not predict a signal peptide for these proteins, a signal peptide was predicted with at least one of the following programs: SignalP-3.0 (88), PridiSi, or DeepTMHMM (89) (for GRA71 only if it would start at the 2nd predicted methionine). Overall, GRA32, GRA57, GRA70, and GRA71 display very similar structural architectures, as predicted by Alphafold (Fig. S3). In all cases, double or triple helices found within the N terminus form a probable coil-coil domain, which is connected to one or two globular domains in the C terminus. These similarities imply possible redundant roles in function, as the coiled-coil domain probably drives homo or heterooligomerization with other coil-coil domains, while the globular domains probably act as specific binders of peptides or small molecule. Of note, Foldseek structural similarity searches using *T_m_* alignments of these globular domains (90) against all existing PDB structures and the entire Alphafold/EBI databases did not identify significant homology to proteins with defined functions, suggesting that these domains could have evolved specifically for the purpose of these GRA proteins. The common architecture of these GRAs leads us to wonder if these proteins form heterooligomers as suggested by the immunoprecipitation data or instead are copurified within the same subcellular organelles. AlphaFold2 predictions setup to run with homodimerization or heterodimerization parameters (within CollabFold) on GRA32/70/71 (GRA57 being too big for multimeric predictions) indicate that both homo- and heterocomplexes could be driven by the coiled-coil domains in some of the observed models (Fig. S4A and B). Homodimers almost always display consistent orientations of the globular domains, leading to protruding helical domains, which act as stalks. In the case of the GRA32 homodimer (Fig. S4A), the model appears to have a symmetry plane in between the aforementioned domains, a feature which is often found within many dimeric assemblies. Heterodimers display less consistent assemblies and may reflect lower likelihood or Alphafold2 limitations to predict such big heterocomplexes with limited homology to other structures within the PDB. These predictions also fall short of addressing the true stoichiometry of these complexes, as higher order oligomers cannot be predicted by Alphafold2 due to excessive protein size limitations, though one could speculate that they also rely on comparable coiled-coil domain interactions.

10.1128/mbio.00060-23.8 FIG S3 Common structural features within GRA32, GRA70, GRA71 and GRA57. Using the best ranked models of Alphafold2, distance error scoring heatmaps define the subdomain features within (A) GRA32/TGME49_212300, (B) GRA70/TGME49_249990, (C) GRA71/TGME49_309600, and (D) GRA57/TGME49_217680. In all cases, an independent coiled-coil domain (in green) can be found in the N terminus followed by one or several globular domains (magenta and red) as displayed both schematically and structurally in a cartoon fashion. Coiled-coil predictions histogram curves obtained from the PRABI-GERLAND server are displayed above the schematic representation of the protein. A score of 0 to 1 is displayed as a function of peptide windows of 14, 21 and 28 residues colored, respectively, in red, green, and blue. Download FIG S3, TIF file, 6.8 MB. Copyright © 2023 Krishnamurthy et al. 2023 Krishnamurthy et al. https://creativecommons.org/licenses/by/4.0/ This content is distributed under the terms of the Creative Commons Attribution 4.0 International license.

10.1128/mbio.00060-23.9 FIG S4 CollabFold/AlphaFold2 multimeric predictions displaying coiled-coil mediated interfaces. (A) GRA32/TGME49_212300, GRA70/TGME49_249990 and GRA71/TGME49_309600, run with a forced homodimerization. Monomer 1 and 2 are colored in salmon and purple, respectively. (B) Heterodimerization predictions between GRA70/TGME49_249990 (yellow), GRA71/TGME49_309600 (dark cyan), and GRA32/TGME49_212300 (magenta). In all cases, the model displaying the most consistent dimerization interface was selected out of 5 predictions. Download FIG S4, TIF file, 7.3 MB. Copyright © 2023 Krishnamurthy et al. 2023 Krishnamurthy et al. https://creativecommons.org/licenses/by/4.0/ This content is distributed under the terms of the Creative Commons Attribution 4.0 International license.

### Host cell stimulation by IFNγ affects parasite fatty acid and cholesterol homeostasis independent of GRA57, GRA70, or GRA71.

**FIG 4:**
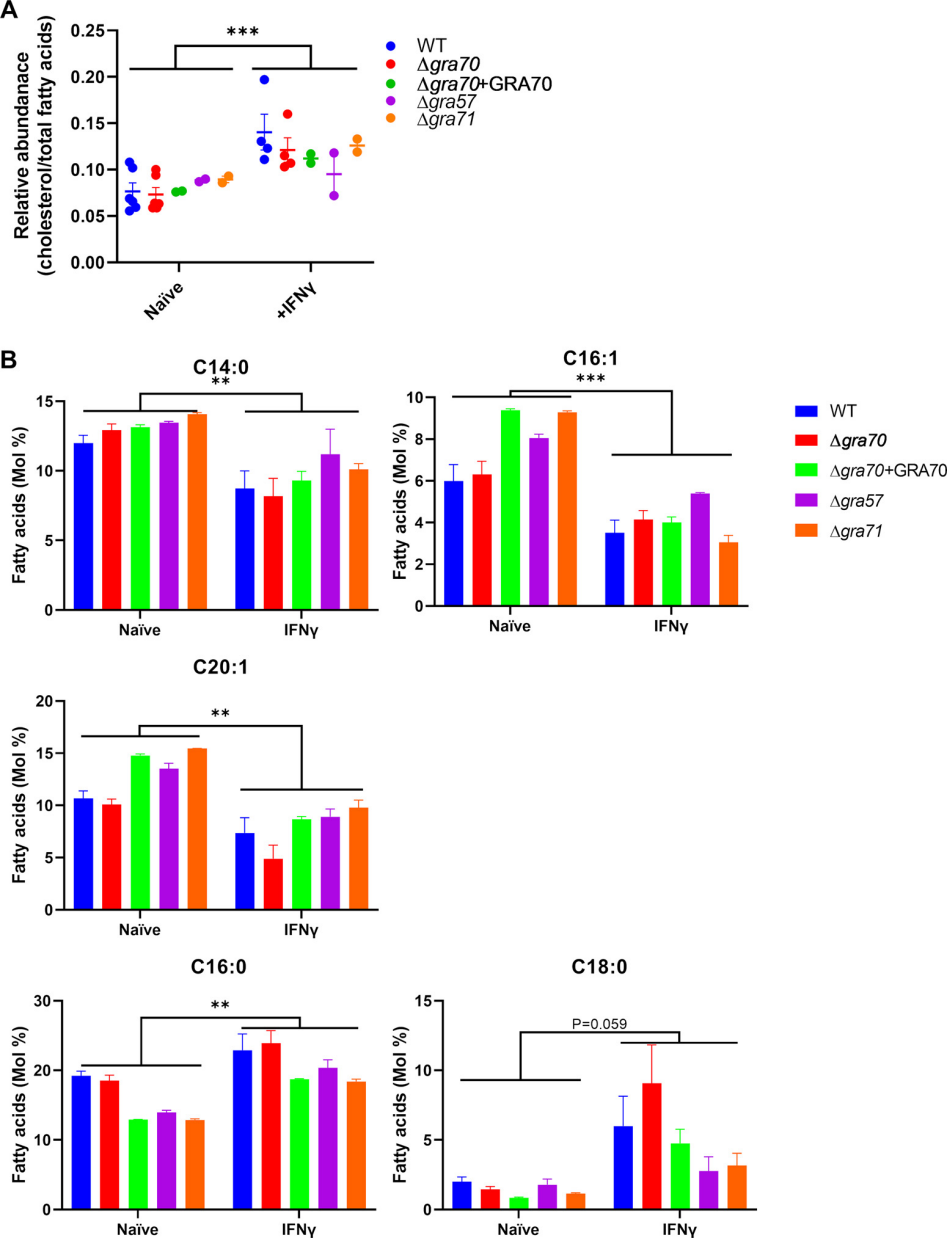
Lipidomic analyses on Δ*gra57*, Δ*gra70*, and Δ*gra71* using GC-MS-based approaches. (A) Lipidomics analysis comparing relative abundance of cholesterol over total fatty acids in WT and indicated knockout and complemented parasites grown in naive or IFNγ-stimulated HFFs. Indicated are means ± SEM (*n* = 2 to 4). *P* values were calculated using 2-way ANOVA with Sidak’s multiple-comparison test. (B) Fatty acid composition in Mol %. *P* values were calculated with a mixed-effects model with Sidak’s multiple-comparison test. *n* = 6 to 8 for WT and Δ*gra70*, *n* = 2 for Δ*gra57*, Δ*gra71*, and Δ*gra70*+GRA70. *, *P* < 0.05; **, *P* < 0.01; ***, *P* < 0.001.

The coiled-coil domain of GRA70 has some homology to the coiled-coil domain of Apolipophorin III (apoLp-III) (Pfam, E value = 0.0014), which functions in transport of diacylglycerol, possibly because they both form amphipathic alpha-helices. Apolipoproteins bind to lipids and are involved in lipid metabolism and reverse cholesterol transport (91). IFNγ and IFNβ upregulate Cholesterol 25-hydroxylase (*CH25H*) leading to the production of 25-hydroxycholesterol (25HC), which inhibits host cell cholesterol metabolism. 25HC has been shown to restrict viruses (92) and bacteria (93, 94) through a variety of mechanisms. Similarly, our analysis of transcriptomic data of several rodent host cell types revealed downregulation of almost all genes involved in cholesterol metabolism after treatment with IFNγ (58). Analysis of transcriptomic data of IFNγ-stimulated human fibroblasts (95) indicated that the cholesterol homeostasis and fatty acid metabolism pathways were significantly modulated in this cell type (Fig. S5A). Furthermore, IFNγ and 25HC upregulated the number of lipid droplets in HFFs, indicating that IFNγ modulated HFF lipid metabolism (Fig. S5B and C). Because *Toxoplasma* genes that determine fitness in IFNγ-stimulated HFFs were enriched for sterol biosynthesis, we took a quantitative mass spectrometry-based lipidomics approach to examine if cholesterol levels are affected in Δ*gra57*, Δ*gra70*, and Δ*gra71* parasites compared to wild-type and complemented parasites in IFNγ-stimulated HFFs. We observed an overall significant increase in cholesterol content when comparing parasites grown in naive HFFs versus grown in IFNγ-stimulated HFFs (Fig. 4A). However, no significant differences in cholesterol between knockout and wild-type/complemented parasites could be detected. To determine whether these knockout lines were further affected at any other lipid content, we quantified the total fatty acid contents in each of the lines with or without IFNγ stimulation. The content of C14:0, C16:1, and C20:1 that are more typically made *de novo* by the parasite (96) was significantly decreased under IFNγ stimulation whereas, FA species like C16:0 and C18:0, which are usually scavenged from the host, were significantly increased (Fig. 4B). However, no significant differences in FA species between knockout and wild-type/complemented parasites could be detected. Thus, although parasite lipid homeostasis is significantly affected by IFNγ, this seems to be independent of GRA57, GRA70, and GRA71.

10.1128/mbio.00060-23.10 FIG S5 IFNγ modulates lipid metabolism in human fibroblasts. (A) Gene set enrichment analysis using the Hallmark pathway database was used on transcriptomic data of human fibroblasts (95) that were stimulated for 5 h with 1,000U IFNγ. (B) Representative fluorescence microscopy images of neutral lipids detected by BODIPY 493/503 (green) in naïve HFFs and HFFs stimulated for 24 h with 100U IFNγ, 20 μm Oleic Acid or 2 μm 25-HydroxyCholesterol (25HC). DNA was stained by Hoechst. (C) Lipid droplets (LD) with a size between 0.5 and 2 μm were quantified in multiple images and the total number of LD in each image are plotted (small dots). Triangles represent the average LDs in the images of one biological replicate (*n* = 3). One way-ANOVA with Holm-Šídák's multiple-comparison test was used to determine statistical significance between averages in naïve versus stimulated HFFs. Download FIG S5, TIF file, 7.7 MB. Copyright © 2023 Krishnamurthy et al. 2023 Krishnamurthy et al. https://creativecommons.org/licenses/by/4.0/ This content is distributed under the terms of the Creative Commons Attribution 4.0 International license.

### Deletion of *GRA66* or *GRA70* leads to early parasite egress in IFNγ-stimulated HFFs.

**FIG 5:**
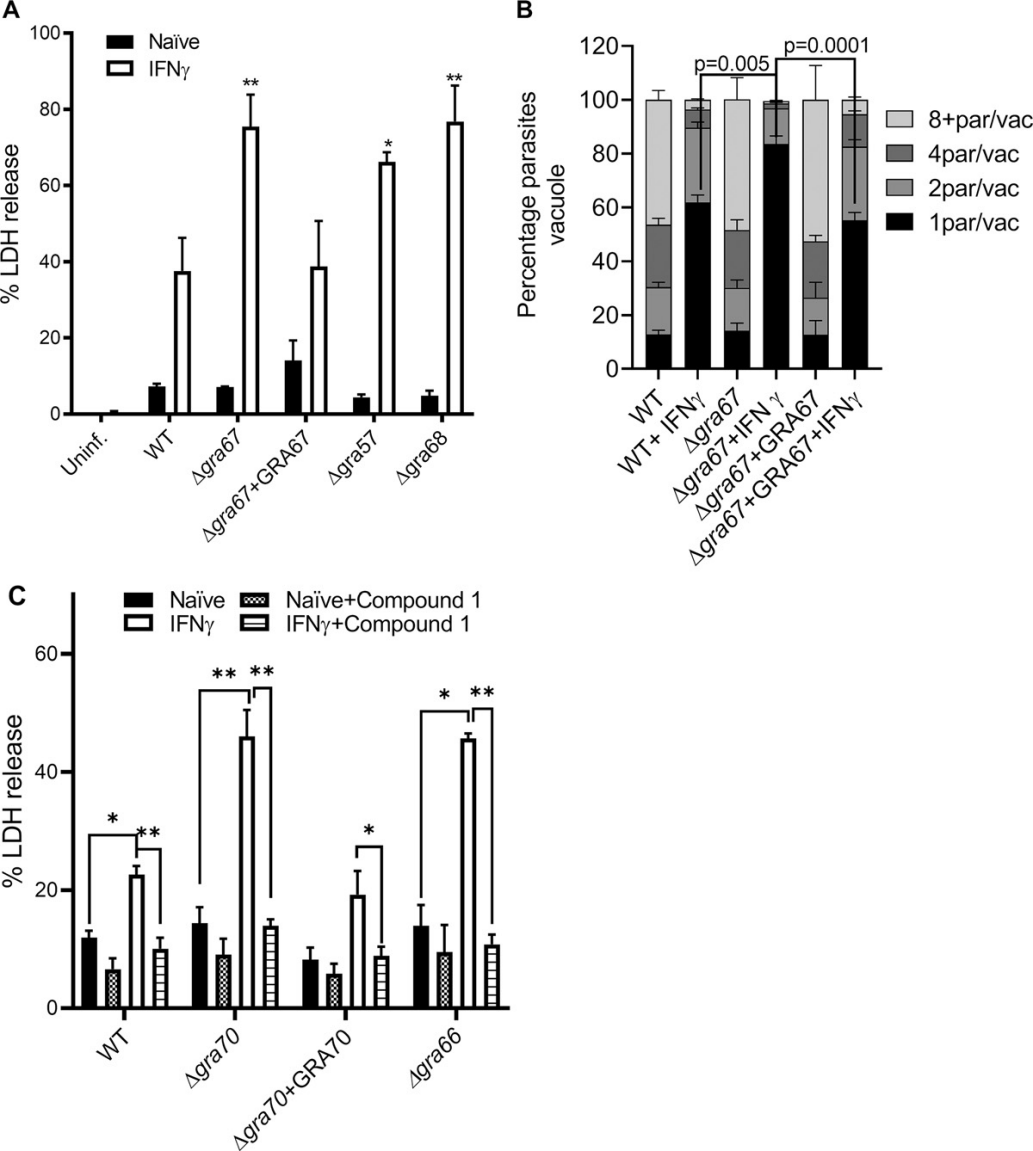
Infection of IFNγ-stimulated HFFs with candidate gene knockout parasites leads to increased host cell death due to early parasite egress. HFFs were prestimulated with IFNγ (10 to 20 U/mL) and subsequently infected with indicated parasite strains (in RH background) for 24 h, after which (A) LDH release in the supernatant was measured. Plotted is the % LDH release compared to maximal LDH release (after triton treatment of cells). (B) The number of parasites/vacuole were counted. (C) As in panel A, but 6 h after infection, 1 μM the PKG inhibitor Compound 1 was added. Indicated are means ± SEM from 3 (A, B, and C for Δ*gra70*+GRA70, and Δ*gra66*) or 6 biological replicates (C for WT and Δ*gra70*). Two-way ANOVA followed by Tukey’s multiple-comparison test was used for statistical analysis. *, *P* < 0.05; **, *P* < 0.01.

We previously showed that *Toxoplasma* infection of IFNγ-stimulated HFFs resulted in host cell death by an unidentified mechanism which caused early parasites egress without replication (31). Because several of the *Toxoplasma* genes that determine fitness in IFNγ-stimulated HFFs have previously been shown to be involved in regulating egress, we determined if the GRA32-like genes might regulate parasite egress. Parasite egress is normally concomitant with host cell death. As previously shown (31), we observed a significant increase in cell death in IFNγ-stimulated HFFs infected with *Toxoplasma* compared to unstimulated HFFs, consistent with parasite egress. Cell death of IFNγ-stimulated HFFs infected with Δ*gra57*, Δ*gra70*, or Δ*gra71* parasites was significantly increased compared to wild-type infected IFNγ-stimulated HFFs (Fig. 5A). Counting of the number of parasites/vacuole 24 hours postinfection (hpi) showed that there were significantly more single parasites per vacuole for the Δ*gra70* parasites in IFNγ-stimulated cells compared to wild-type and complemented parasites (Fig. 5B). These data are consistent with early egress of Δ*gra70* parasites in IFNγ-stimulated HFFs. Indeed, inhibition of parasite PKG, which is essential for parasite egress, with Compound 1 (97) inhibited the enhanced host cell death after infection with Δ*gra70* parasites (Fig. 5C). Similar results were observed after infection with Δ*gra66* parasites. Overall, these data are consistent with a role for GRA57, GRA66, GRA70, and GRA71 in preventing early egress, specifically in IFNγ-stimulated HFFs.

## DISCUSSION

IFNγ stimulates a variety of effector mechanisms that restrict *Toxoplasma* (37). Despite that, the parasite establishes lifelong chronic infections by secreting GRAs and ROPs into the host cell that can inhibit host immunity. Many parasite effectors determine parasite fitness specifically in IFNγ-stimulated murine cells (38) because they target the IRGs, which are not present in humans. In contrast, the *Toxoplasma* effector TgIST, which inhibits IFNγ-induced STAT1 signaling, functions in both rodent and human cells (48, 49). How *Toxoplasma* survives after infection of human cells that were previously stimulated with IFNγ is largely unknown. Here, we performed genome-wide loss-of-function screens and identified multiple *Toxoplasma* genes important for parasite fitness specifically in IFNγ-stimulated HFFs. Many of these *Toxoplasma* genes are predicted to be involved in regulating egress, response to oxidative stress, and nutrient acquisition. Parasite genes that were previously shown to affect GRA export beyond the PV (81) were not in the top 2 percentile of hits in our screen, except for *ROP17*, *MYR3*, and *GRA45*. It is possible that ROP17 and MYR3 not only affect GRA export but, like GRA45, also affect the correct localization of GRAs such as GRA23 (a hit in our screen) to the PVM (58).

In this study, we mostly focused on three genes (*GRA57*, *GRA70*, and *GRA71*) that encode proteins with homology to GRA32 (GRA32-like). We confirmed that GRA57, GRA70, and GRA71 are important for parasite fitness in IFNγ-stimulated HFFs, and based on our mass spectrometry data, these proteins likely interact with each other and GRA32 and might function in a tetrameric complex. This is supported by a recent paper in which a coelution strategy was used to generate a genome-scale physical protein interaction network for *Toxoplasma* and which identified a protein complex that contained GRA32, GRA70 and GRA71, among others (98). Our data show that infection of IFNγ-stimulated HFFs with parasites in which these GRA32-like proteins were knocked out led to significantly more host cell death compared to infection with wild-type parasites. This host cell death could be inhibited by preventing parasite egress with the PKG inhibitor Compound 1, indicating that cell death was caused by parasite egress. In the last years, multiple other GRAs have been shown to be involved in the regulation of timing of egress. For example, Δ*gra41* and Δ*gra22* parasites have a premature egress phenotype, while knockouts of the *Toxoplasma* lecithin:cholesterol acyltransferase (*LCAT*) and diacylglycerol kinase (*DGK2*) have a defect in natural egress (69, 99–101). It is currently unclear how exactly GRA57, GRA70, and GRA71 regulate parasite egress in IFNγ-stimulated HFFs. One potential clue is that GRA66, which was immunoprecipitated with GRA57 and affects egress in IFNγ-stimulated HFFs, encodes a NAPE-PLD. PLDs can usually produce phosphatidic acid, a key lipid species shown to be involved in regulating parasite egress, and suggested to be generated in the PV lumen, notably via the action of TgDGK2 on diacylglycerol (69). GRA32-like proteins could potentially regulate the activity of GRA66 or another route to generate phosphatidic acid in the PV lumen. RARRES3, an IFNγ-induced host protein that was recently shown to affect parasite egress in IFNγ-stimulated human cells (35), has predicted dual phospholipase and acyltransferase activity suggesting that modification of both host and parasite lipids can play a role in regulating parasite egress.

It is currently unclear why the GRA32-like proteins specifically affect parasite egress in IFNγ-stimulated HFFs. A potential clue is that the parasite lipid profile differed depending on if they were grown in naive or IFNγ-stimulated HFFs. Human cells, including HFFs, acquire cholesterol *in vitro* either by *de novo* synthesis or from exogenous sterols by low-density lipoprotein (LDL)-mediated endocytosis or from esterified cholesterol (102). Host cell *de novo* synthesis of cholesterol is not essential for parasite growth and remains unchanged upon infection with the type I strain of *Toxoplasma* (102), even though some genes in the mevalonate pathway are upregulated upon HFF infection by *Toxoplasma* (103). It remains unclear if upon infection with *Toxoplasma*, the cholesterol levels within the host cells are affected in IFNγ-stimulated HFFs. However, we measured a significant increase in parasite cholesterol levels in IFNγ-stimulated HFFs. The increase in cholesterol within the parasite may be a consequence of increased uptake of LDL-cholesterol by IFNγ-stimulated HFFs from the growth media (102). Several reports (104) show that downregulation of host cholesterol synthesis, which happens in the presence of IFNγ (25), leads to upregulation of the LDL-receptor and of LDL-cholesterol uptake in cells. This might explain the increase in lipid droplets we observed in IFNγ-stimulated HFFs and could also explain why ACAT1-alpha was a hit in our screen, as the increase in parasite cholesterol (and stearic and palmitic acid) in IFNγ-stimulated HFFs likely requires an increased need to store these lipids in lipid droplets to prevent their toxicity. Lipidomics also revealed that scavenged FA were significantly increased upon IFNγ stimulation, while FA typically made by the parasite were decreased. Overall, these data indicate that the altered parasite lipid profile in IFNγ-stimulated HFFs potentially promotes early parasite egress, which is further exacerbated without GRA57, GRA70, or GRA71, possibly because these GRAs regulate specific lipids in the PV lumen or the activity of enzymes (such as GRA66) that can modify lipids. While the manuscript was under review, another group identified *Toxoplasma* genes that specifically determine fitness in IFNγ-stimulated HFFs by using CRISPR screens targeting 253 genes encoding secreted *Toxoplasma* proteins (105). GRA57, GRA70, and GRA71 were also major hits in that screen and major interaction partners in coimmunoprecipitation experiments. Future experiments are required to determine the exact mechanism by which the genes identified in our screen determine parasite fitness in IFNγ-stimulated HFFs.

## MATERIALS AND METHODS

### Cell and parasite culture.

Human foreskin fibroblasts (HFFs) were cultured as previously described (72). Briefly, HFF were grown in Dulbecco’s modified Eagle medium (DMEM) containing 10% fetal bovine serum (FBS), 100 U/mL penicillin/streptomycin (Gibco 15140-122), 2 mM l-glutamine (Gibco 25030-081), and 10 μg/mL gentamicin (Gibco 15710-064). For plaque assays and growth competition assays, HFFs were seeded into 24-well and 6-well plates, respectively, and infected 3 days later. HFFs used for indirect immunofluorescence assays were seeded on 12-mm glass coverslips (VWR 48366-251) in 24-well plates 2 days before infection. Parasites were maintained on confluent HFF monolayers in DMEM with 1% FBS and 100 U/mL penicillin/streptomycin. All parasite transfections were performed as previously described (106).

### Analysis of the loss-of-function screens to identify parasite genes that determine fitness in IFNγ-stimulated HFFs.

The genome-wide loss-of-function screen in *Toxoplasma* using CRISPR/Cas9 gene editing technology was performed as described previously (54, 55) (Fig. 1A). Briefly, a library of single guide RNAs (sgRNAs) containing 10 guides against 8,156 *Toxoplasma* protein coding genes were cloned in the pU6 sgRNA expression vector containing the dihydrofolate reductase (DHFR) resistance cassette (pU6-DHFR). Ten cuvettes, each with 1 × 10^8^ RH-Cas9-expressing parasites, were transfected with 100 μg of AseI linearized dialyzed pU6-DHFR vector, and parasites with integrated plasmids were selected using 1 μM pyrimethamine. The transfectants (5  ×  10^7^ parasites per 150 mm tissue culture dish [Corning no. 353025]; 20 dishes total) were consecutively passaged for four to five rounds (2 × 10^7^ parasites/dish; 10 dishes total) in HFFs with DMEM containing 1 μM pyrimethamine to select against parasites that integrated plasmids with sgRNAs that targeted fitness-conferring genes in unstimulated HFFs. At each passage, parasite pellets with ~1 × 10^8^ parasites were collected for DNA isolation and sgRNA amplification by PCR using the primers listed in Table S1 for Illumina sequencing. After four passages in HFFs, we transferred 2 × 10^7^ parasites per tissue culture dish (10 dishes total) for four passages in either naive or HFFs prestimulated with 10 to 20 units human IFNγ. Because the potency of IFNγ can differ from batch to batch, the units of IFNγ used were based on getting 30% growth inhibition of wild-type parasites. We compared sgRNA abundance in parasite pellets isolated from naive and IFNγ-stimulated HFFs from the different passages. The abundance of each individual sgRNA was normalized to the total number of reads, followed by log_2_ transformation. The sgRNAs with 0 reads were replaced by the 90% of the lowest value in that sample (Table S2). To assess the change in abundance of sgRNAs from the starting point to the endpoint of the experiment, an average phenotype score, which we refer to as the fitness score, was calculated as the log_2_ fold change of the top 5 sgRNA abundance between parasites isolated from the endpoint versus library (Table S3). The raw reads of sgRNAs from IFNγ-stimulated and naive HFFs were used to generate a list of negatively selected genes with *P* values for each data set using MaGeCK analysis (107). Absolute Cohen’s *d* values were used to calculate the effect size (108) (Table S3).

10.1128/mbio.00060-23.1 TABLE S1 List of the primers, oligonucleotides for sgRNA constructs, and information on the antibodies used. Download Table S1, XLSX file, 0.02 MB. Copyright © 2023 Krishnamurthy et al. 2023 Krishnamurthy et al. https://creativecommons.org/licenses/by/4.0/ This content is distributed under the terms of the Creative Commons Attribution 4.0 International license.

10.1128/mbio.00060-23.2 TABLE S2 The RAW_READ_FREQ_PHENOTYPES contains the number of reads for each specific sgRNA, which was subsequently converted into phenotype scores as described (55). Download Table S2, XLSX file, 6.8 MB. Copyright © 2023 Krishnamurthy et al. 2023 Krishnamurthy et al. https://creativecommons.org/licenses/by/4.0/ This content is distributed under the terms of the Creative Commons Attribution 4.0 International license.

### Generation of knockout, complemented, and transgenic parasites.

The list of sgRNAs used to generate each individual knockout parasite is listed in Table S1. All the parasite gene knockouts were generated using the RH-Luc/Δ*hxgprt* strain (56). Individual sgRNAs were inserted into the BsaI (NEB) site in vector pU6-Universal (54). Parasites were transfected with pU6-Universal containing the sgRNA for the gene of interest (GOI) to generate Cas9-directed double-stranded DNA breaks, which were then repaired using a template containing a GFP-HXGPRT cassette obtained from EcoRV (NEB) linearized pTKO plasmid (109). The transfectants with the GFP-HXGPRT cassette at the specific Cas9 cut site were selected with 50 μg/mL mycophenolic acid (MPA) (Millipore 89287) and 50 μg/mL xanthine (Xan) (Millipore X3627) (58). Single clones of knockout parasites were isolated by limiting dilution following three rounds of drug selection with MPA-Xan. The primers used to screen the individual knockout parasites for insertion of the repair cassette in the forward or reverse orientation are listed in Table S1.

Parasites with C-terminal endogenously tagged genes with a 3xHA epitope were generated using ligation independent cloning (LIC) in the RHΔ*ku80*Δ*hxgprt* parasite strain (110). The primers used for amplification of C-terminal ends of the GOI without the last stop codon are listed in Table S1. After three rounds of selection with pyrimethamine, single parasite clones were isolated by limiting dilution and checked for presence of the epitope tag using indirect immunofluorescence assay (IFA) with anti-HA antibody (Roche).

Gibson assembly (111) using the NEB HiFi assembly kit was used to generate a vector with C-terminal triple-HA epitope tag in the pUC19 vector backbone (112) to complement TGGT1_249990 back into the Δ*249990* parasites. The 5′ upstream and 3′ downstream fragments of TGGT1_249990 were amplified from the genomic DNA of the parental wild-type parasite strain using primers in Table S1. The coding sequence (CDS) was amplified from the cDNA of the type I parental strain using primers in Table S1. Sanger sequencing was used to check the integrity of the 5′ UTR, CDS, and stop codon after the epitope tag. Next, 10 μg of NcoI (NEB) linearized pTKO-DHFR plasmid along with 50 μg of NdeI (NEB) and SacI (NEB) linearized TGGT1_249990 complementation vector in the pUC19 vector backbone was used to transfect Δ*249990* parasites. Random integration of the gene was promoted using the DHFR cassette and complemented strains were selected using pyrimethamine. Following limiting dilution, single clones were screened by IFA as described below.

### Growth competition assay.

To mimic the results from the genome-wide loss-of-function screen, the growth medium was supplemented with 1 mM sodium pyruvate (Gibco 11360-070), 1 ×  nonessential amino acids (Gibco 11140-076), and 10 mM HEPES (Gibco 15630-080) in addition to l-glutamine, gentamicin, penicillin/streptomycin and 10% FBS. The media in 6-well plates containing confluent monolayers of HFFs was changed to media with or without 10 U/mL human IFNγ. On the day of infection, wild-type (GFP-negative) and knockout parasites (GFP-positive) for the competition were harvested and counted, and 5 × 10^5^ of each parasite strain was mixed to infect 6-well plates (with or without IFNγ). At each passage with and without IFNγ, 5 × 10^5^ parasite mix was used for infection. Plaque assays were performed as described above to determine the ratio of knockout: total parasites at passages 0, 1, 3, and 6. The total number of plaques were counted using a brightfield microscope and GFP-expressing knockout parasite plaques were counted using epifluorescence. All graphs were plotted using GraphPad prism and two-way ANOVA followed by Sidak’s multiple-comparison test was used to test for significance from three biological replicates, with each plaque assay in triplicate wells per condition.

### Plaque assay.

Confluent monolayers of low-passage HFFs in 24-well plates were used for plaque assays, as previously described (113). DMEM media with 10% FBS with or without human IFNγ (20 U/mL, AbD Serotec) was used to replace the media in the assay plate 24 h before infection. Seventy-five parasites were used to infect each well of the 24-well plate containing naive or IFNγ-stimulated HFFs. On day five postinfection (pi), plaques were imaged using a 4 ×  objective Nikon TE2000 inverted microscope equipped with a Hamamatsu ORCA-ER digital camera. Percentage plaque loss was calculated using the following formula: ([number of parasite plaques in unstimulated HFFs –number of parasite plaques in IFNγ stimulated HFFs] / number of parasite plaques in unstimulated HFFs *100). All experiments were performed at least 3 times with triplicate wells for each condition.

### Indirect immunofluorescence.

All IFAs were performed using previously published protocols (109). Confluent HFF monolayers grown in 12-mm coverslips were used to infect with parasites for IFA. Depending on the primary antibody (listed in Table S1), the cells were fixed for 20 min either with 4% paraformaldehyde (PFA) or for 5 min on ice with cold 100% methanol and blocked for 30 min in a buffer containing 3% BSA (Sigma A9647), 5% goat serum, 0.02% Triton X-100, and 0.01% sodium azide in PBS. Primary antibodies were diluted in blocking buffer and used to stain cells overnight at 4°C. After 3 washes with PBS, secondary antibody diluted (Table S1) 1:3,000 was used for 1 h at room temperature along with Hoechst 33258 (Invitrogen) at 1:2,000 to stain DNA. Coverslips were washed 5 times with PBS and mounted on glass slides using Mowiol (Sigma).

### Parasites per vacuole counting.

Parasites were harvested and filtered and a multiplicity of infection (MOI) of 0.5 of each parasite strain (RH-Luc/Δ*hxgprt*, RH-LucΔ*249990*, and RH-LucΔ*249990 *+ 249990) was added to coverslips in 24-well plates containing a monolayer of HFFs with or without stimulation with 20 U/mL IFNγ for 24 h before infection. The plates were centrifuged at 900 *g* for 3 min and incubated at 37°C in a CO_2_ incubator. At 30 min postinfection, the uninvaded parasites were washed away from the coverslips using PBS. The coverslips were fixed at 24 hpi and processed for IFA as mentioned above using GRA7 and SAG1 antibodies. Twenty-five fields per coverslip were used for parasite enumeration and data were plotted using GraphPad Prism.

### Immunoprecipitation.

Ten 150-mm tissue culture dishes with confluent monolayers of HFFs were used for infection with each parasite strain at an MOI of 3. All parasite strains used for immunoprecipitation had the gene of interest (GOI) with a C-terminal 3xHA epitope tag. Parasites were harvested and pellets were lysed using lysis buffer as described in (106) for 30 min on ice. After centrifugation, lysates were incubated with Pierce magnetic beads coupled with antibodies against the HA epitope (cat no. 88837) at 4°C overnight. The beads were subsequently washed three times with lysis buffer and processed for peptide identification by LC-MS/MS mass spectrometry following trypsin digestion. To identify proteins that specifically interact with TGGT1_249990 or GRA57, a minimum of 7 unique peptide count cutoff was applied to all immunoprecipitations and only hits with a 4-fold enrichment of peptide counts compared to control immunoprecipitations (TGGT1_226380/GRA35, TGME49_275470/GRA15 [46] and the GRA TGGT1_263560 [58]) were included.

### Luciferase and cell viability assays.

Luciferase assays along with cell viability assays were performed as previously described (113). Briefly, HFFs were seeded in 96-well plates in complete media. The following day, the medium was replaced with fresh media with or without IFNγ for 24 h before infection with parasites. On the day of the experiment, parasites were harvested to infect HFFs at an MOI of 1, 2, and 3. In parallel, plaque assays with individual parasites were performed to determine the actual MOI. Parasite growth was measured by luciferase assay and host cell viability was determined using lactate dehydrogenase (LDH) from the culture supernatant 24 hpi using a microplate reader (Molecular device M2e, CA, USA). Matched parasite MOIs (as determined by plaque assay) were plotted from three biological replicates, each condition in triplicates using GraphPad Prism.

### Egress assay.

HFFs were seeded in 96-well plates in complete media. The following day, the medium was replaced with fresh media with or without IFNγ for 24 h. On the day of the experiment parasites were harvested to infect HFFs at an MOI of 2 and 3. In parallel, plaque assays with individual parasite strains were performed to determine the actual MOI. After 6 h of infection, the 96-well plates were washed and 1 μM compound 1 was added in media with and without IFNγ. Host cell death was used as a measure of parasite egress and was determined 24 hpi by measuring LDH in the culture supernatant.

### Protein motif analysis and alphafold structure predictions.

To identify protein motifs, the MyHits database and profile-HMM database were searched with the amino acid sequences of the *Toxoplasma* proteins (114, 115). Structural modeling was performed using the Alphafold2 algorithm (116). When available, models were taken from the Alphafold/EBI repository. For larger models or oligomeric predictions, Alphafold was run through the Collabfold Linux environment (117) running on an Nvidia A5000 graphics card. Coil-coil predictions were run on the PRABI-GERLAND server while protein structure visual representations were performed on ChimeraX.

### Lipidomics analysis.

Lipidomics analysis was performed using previously published protocols (96) with the following modifications. The parasites were grown for 48 h or until completely extracellular in confluent monolayers of HFF in flasks (175 cm^2^) that were or not prestimulated for 24 h with human IFNγ (20 U/mL, AbD Serotec). Intracellular tachyzoites (1  ×  10^7^ cell equivalents per replicate) were collected, and host cells were filtered out with a 3-μm pore size membrane. These parasites were metabolically quenched by rapid chilling in a dry ice-ethanol slurry bath and then centrifuged down at 4°C. The parasite pellet thus obtained was washed with ice-cold PBS thrice before transferring the final pellet to a microcentrifuge tube. Total lipids were extracted in chloroform/methanol/water (1:3:1, vol/vol/vol) containing phosphatidylcholine (PC) (C13:0/C13:0 [10 nmol] and C21:0 [10 nmol] as internal standards for extraction) for 1 h at 4°C, with periodic sonication. Subsequently, polar and apolar metabolites were separated by phase partitioning by adding chloroform and water to give the ratio of chloroform/methanol/water, 2:1:0.8 (vol/vol/vol). For lipid analysis, the organic phase was dried under N2 gas and dissolved in 1-butanol to obtain 1 μL butanol/10^7^ parasites.

**(i) Total lipid analysis.** Total lipid was then added with 1 nmol pentadecanoic acid (C15:0) as internal standard and derivatized to give fatty acid methyl ester (FAME) using trimethylsulfonium hydroxide (TMSH, Machenery Nagel) for total glycerolipid content. Resultant FAMEs were then analyzed by GC-MS as previously described (118). All FAMEs were identified by comparing retention time and mass spectra from GC-MS with authentic chemical standards. The concentration of FAMEs was quantified after initial normalization to different internal standards and finally to parasite number.

**(ii) Phospholipid and neutral lipid analysis.** For phospholipid analysis, total lipid extracted (as mentioned above) was separated with 1 nmol PA (C17:0/C17:0) (Avanti Polar lipids) by one-dimensional silica gel high-performance thin-layer chromatography (HPTLC, Merck). For total PL, DAG, TAG, free fatty acids (FFA), and cholesteryl ester (CE) analysis, the total lipid fraction was separated by 1D-HPTLC using hexane/diethyl ether/formic acid, 80:20:2 (vol/vol/vol) as a solvent system. Thereafter, each lipid spot on the HPTLC plate was scraped off and lipids were methanolized with 200 μL 0.5 M methanolic HCl in the presence of 1 nmol pentadecanoic acid (C15:0) as internal standard at 85°C for 3 h. The resulting FAMEs were extracted with hexane and finally analyzed by GC-MS (Agilent).

### Lipid droplet quantification.

HFF cells were grown on coverslips in 24-well plates and confluent monolayers were pretreated (stimulated) with 100 U IFNγ, 20 μm Oleic acid, or 2 μm 25HC (25-Hydroxycholesterol). After 24 h of incubation, cells were washed with 1 ×  PBS followed by incubation with 2 μm BODIPY 493/503 solution (prepared in 1 ×  PBS) for 15 min at 37°C. The cells were washed twice with PBS and fixed with 4% paraformaldehyde in PBS at room temperature for 30 min, then stained with DAPI, and mounted with mounting medium. Cells were imaged on an inverted microscope Nikon (eclipse Ti-S; Nikon) connected to NIS-Elements software (Nikon) using a digital camera (CoolSNAP EZ; Roper Scientific). Fluorescent images were analyzed with NIS-Elements software (Nikon) using a pipeline that identified BODIPY-positive lipid droplets with a size between 0.5 μm to 2 μm. Anything below and above this value was excluded from the analysis. The average number of lipid droplets was counted from multiple different images from 3 biologically independent experiments and data were analyzed by GraphPad prism.

### Gene set enrichment analysis of IFNγ-stimulated human fibroblasts.

Transcriptomic data from IFNγ-stimulated (1,000 U for 5 h) human fibroblasts (95) was downloaded from the GEO database (GSE3920). Average fold changes in gene expression levels between IFNγ-stimulated and unstimulated human fibroblasts were calculated (samples G1 and G2 versus Con1 and Con2) and used as the input for preranked GSEA analysis (119) using the Molecular Signatures Database (MSigDB) hallmark gene set collection (120).

### Data availability.

All the raw data are shown in the supplementary figures and are also available from the corresponding author.
