# Supplementary figures and images for: CRISPR Screens Identify Toxoplasma Genes That Determine Parasite Fitness in Interferon Gamma-Stimulated Human Cells
Source: mBio. 2023 Mar 14;14(2):e00060-23. doi: 10.1128/mbio.00060-23 (PMC10128063; doi:10.1128/mbio.00060-23)

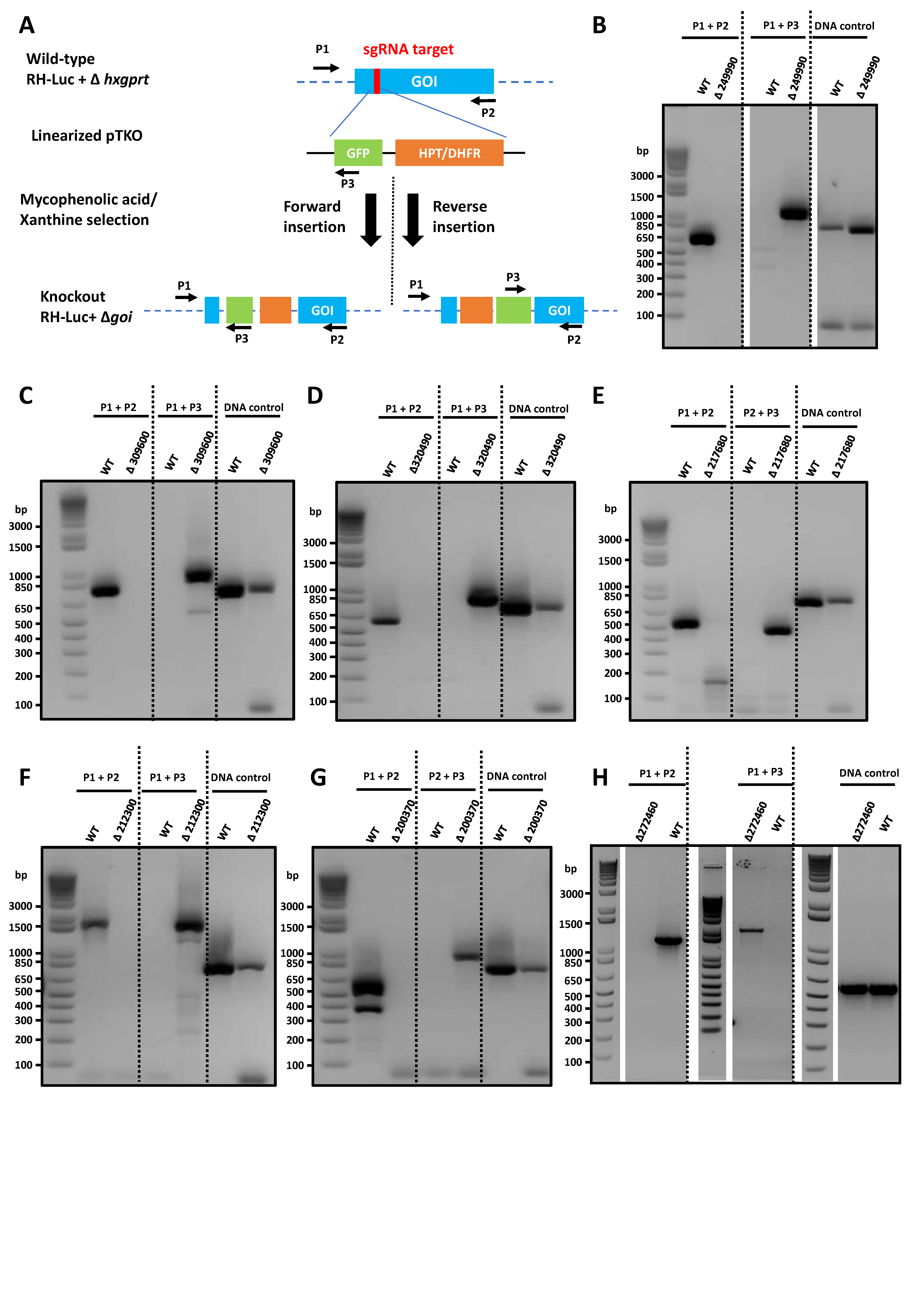

Supplement: FIG S1 [file mbio.00060-23-s0006.tif]

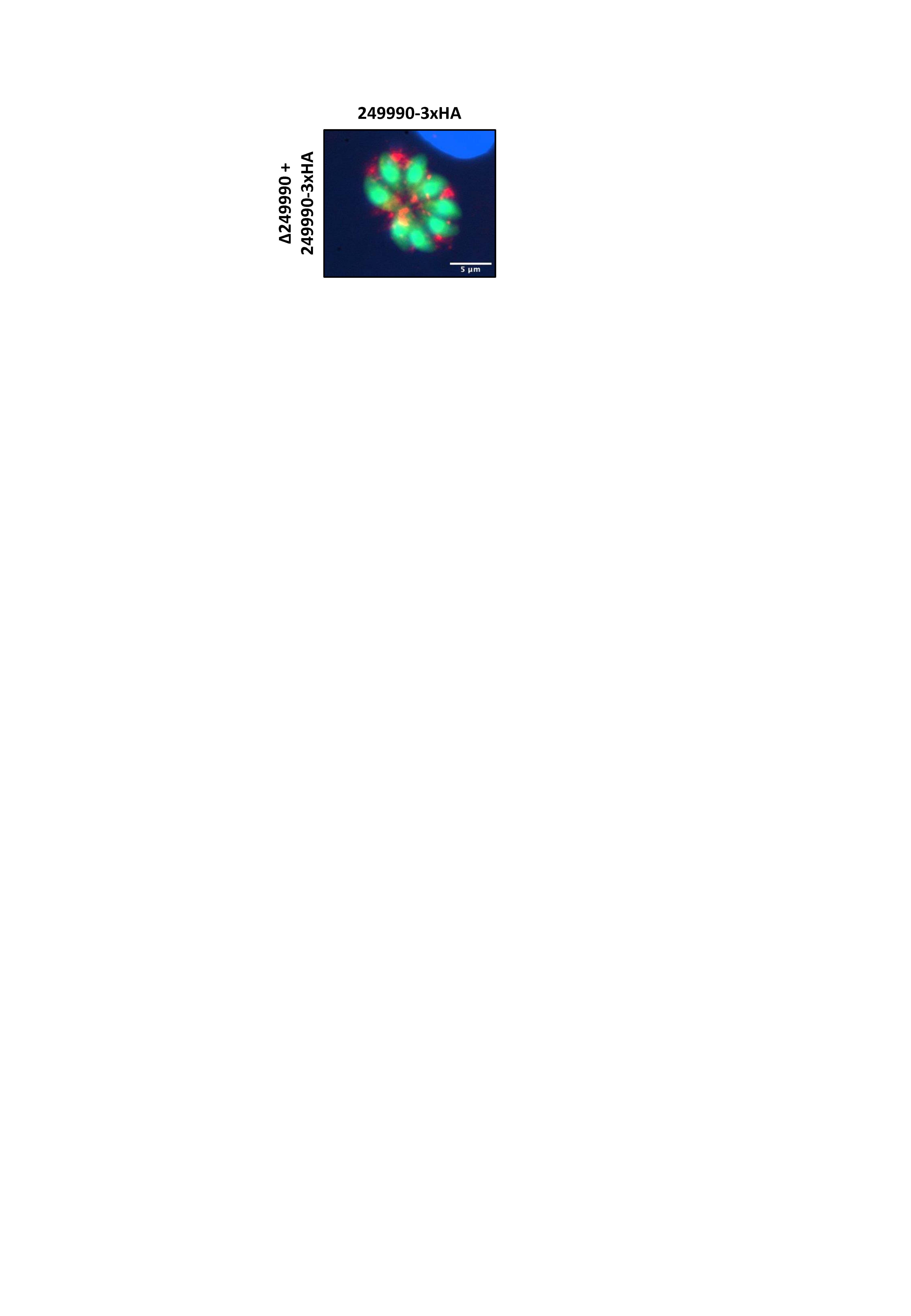

Supplement: FIG S2 [file mbio.00060-23-s0007.tif]

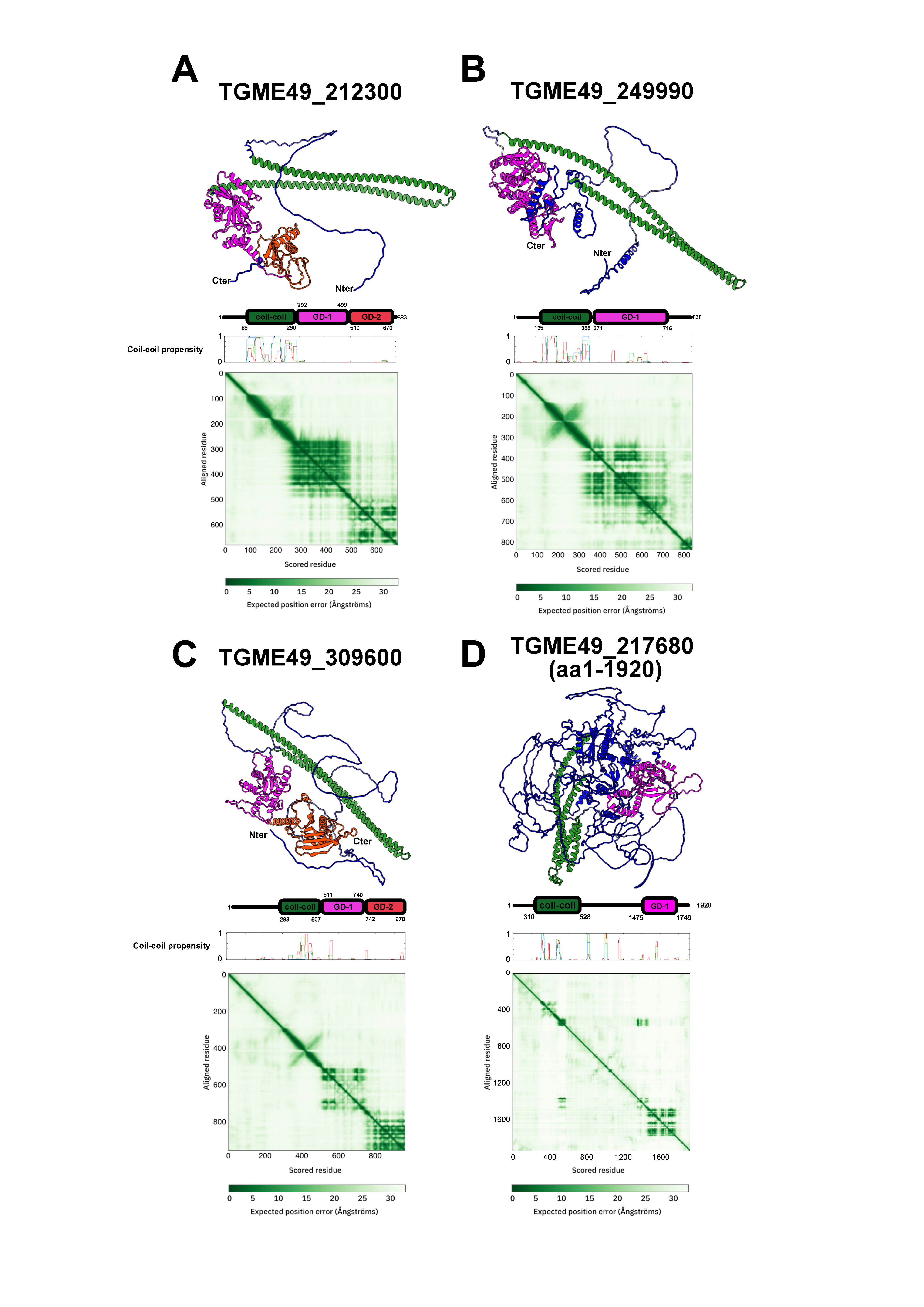

Supplement: FIG S3 [file mbio.00060-23-s0008.tif]

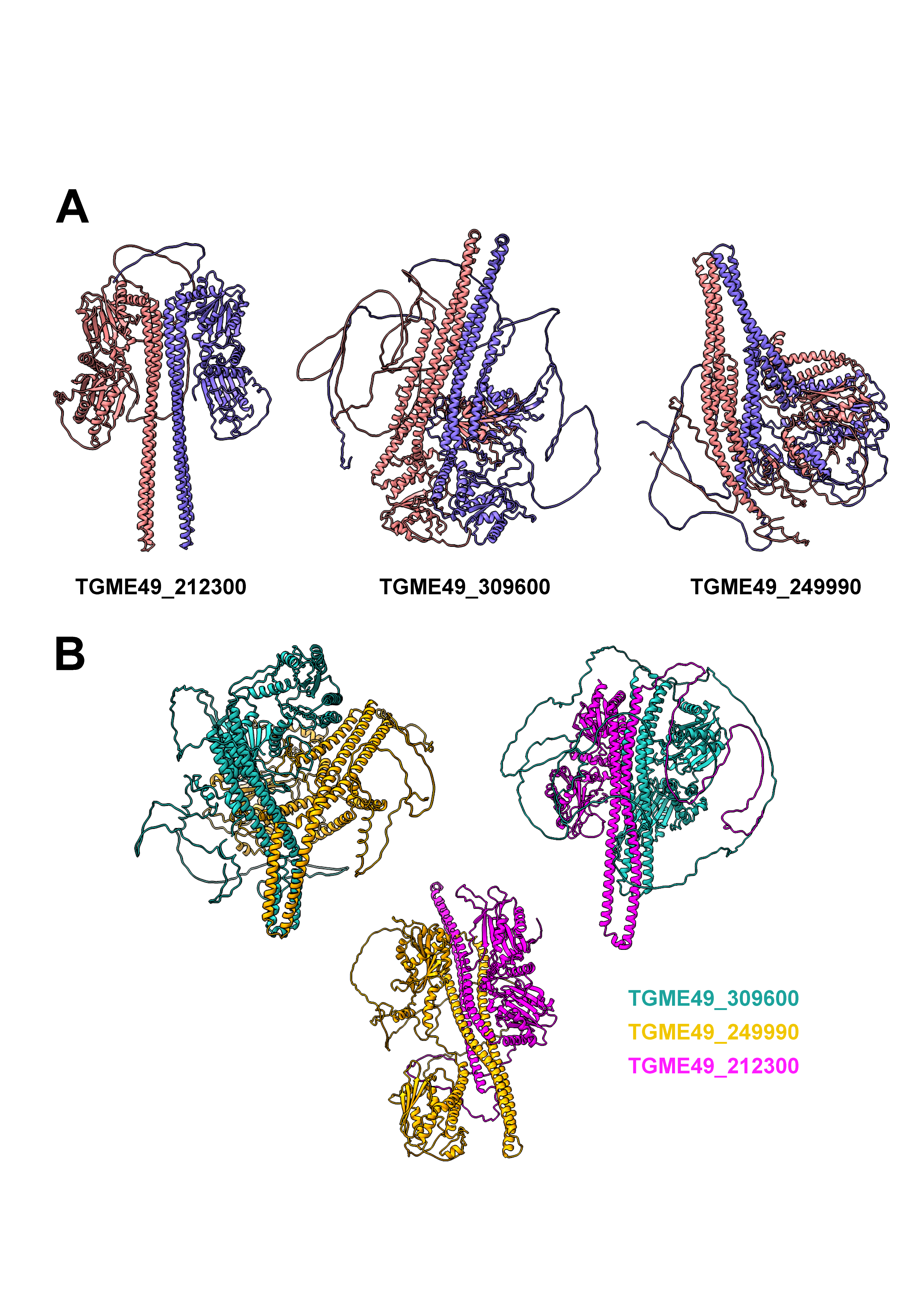

Supplement: FIG S4 [file mbio.00060-23-s0009.tif]

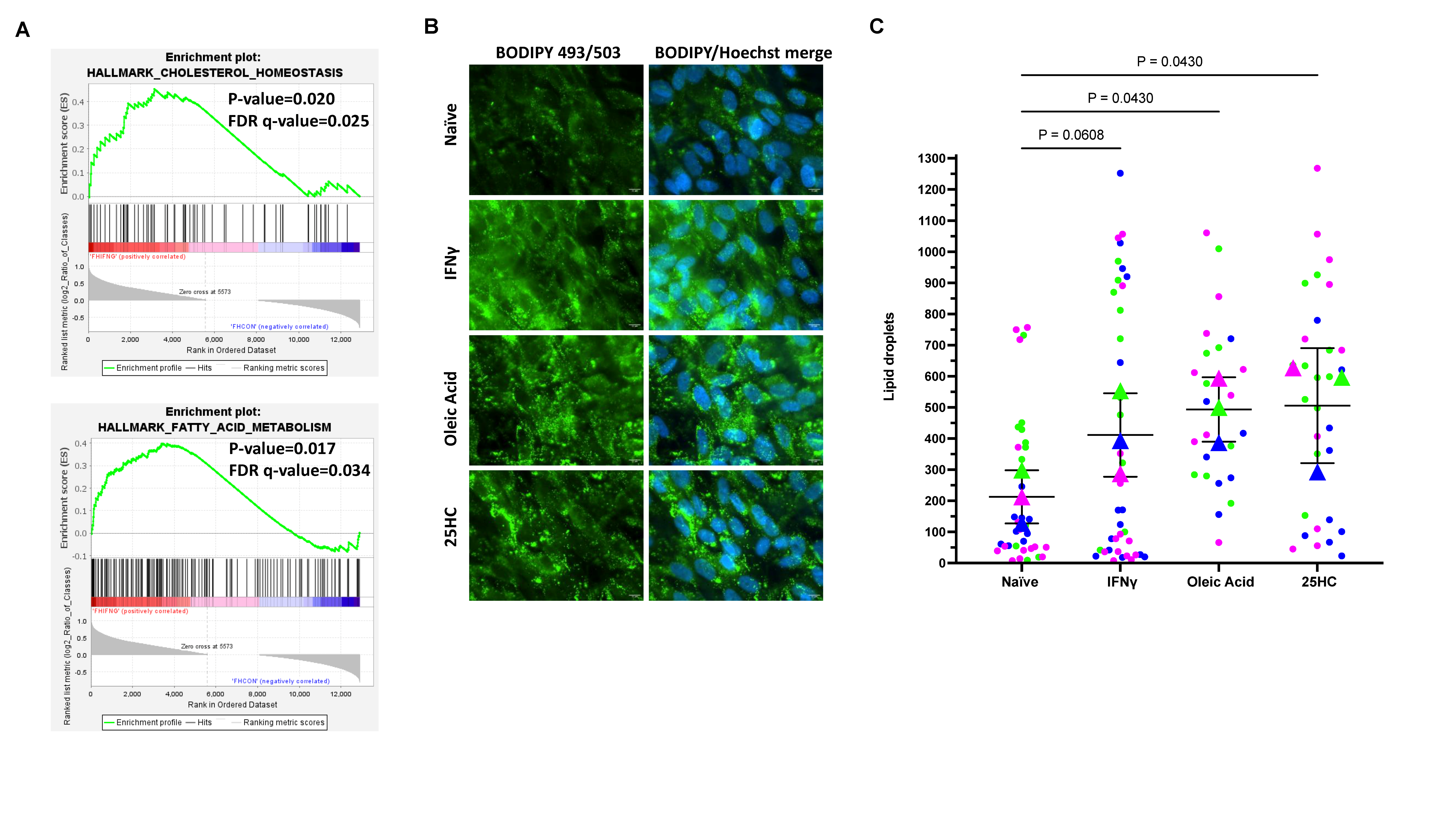

Supplement: FIG S5 [file mbio.00060-23-s0010.tif]
